# Cetacean Acoustic Welfare in Wild and Managed-Care Settings: Gaps and Opportunities

**DOI:** 10.3390/ani11113312

**Published:** 2021-11-19

**Authors:** Paige E. Stevens, Heather M. Hill, Jason N. Bruck

**Affiliations:** 1 Department of Integrative Biology, Oklahoma State University, 501 Life Sciences West, Stillwater, OK 74074, USA; paige.elise.stevens@okstate.edu; 2 Department of Psychology, St. Mary’s University, 1 Camino Santa Maria, San Antonio, TX 78228, USA; hhill1@stmarytx.edu; 3 Department of Biology, Stephen F. Austin University, SFA Station, Nacogdoches, TX 75962, USA

**Keywords:** anthropogenic noise, welfare, cetaceans, marine mammals, managed-care

## Abstract

**Simple Summary:**

Whales and dolphins in managed-care and wild settings are exposed to human-made, anthropogenic sounds of varying degrees. These sounds can lead to potential negative welfare outcomes if not managed correctly in zoos or in the open ocean. Current wild regulations are based on generally broad taxa-based hearing thresholds, but there is movement to take other contextual factors into account, partially informed by researchers familiar with work in zoological settings. In this spirit, we present more nuanced future directions for the evaluation of acoustic welfare in both wild and managed-care settings, with suggestions for how research in both domains can inform each other as a means to address the paucity of research available on this topic, especially in managed-care environments.

**Abstract:**

Cetaceans are potentially at risk of poor welfare due to the animals’ natural reliance on sound and the persistent nature of anthropogenic noise, especially in the wild. Industrial, commercial, and recreational human activity has expanded across the seas, resulting in a propagation of sound with varying frequency characteristics. In many countries, current regulations are based on the potential to induce hearing loss; however, a more nuanced approach is needed when shaping regulations, due to other non-hearing loss effects including activation of the stress response, acoustic masking, frequency shifts, alterations in behavior, and decreased foraging. Cetaceans in managed-care settings share the same acoustic characteristics as their wild counterparts, but face different environmental parameters. There have been steps to integrate work on welfare in the wild and in managed-care contexts, and the domain of acoustics offers the opportunity to inform and connect information from both managed-care settings and the wild. Studies of subjects in managed-care give controls not available to wild studies, yet because of the conservation implications, wild studies on welfare impacts of the acoustic environment on cetaceans have largely been the focus, rather than those in captive settings. A deep integration of wild and managed-care-based acoustic welfare research can complement discovery in both domains, as captive studies can provide greater experimental control, while the more comprehensive domain of wild noise studies can help determine the gaps in managed-care based acoustic welfare science. We advocate for a new paradigm in anthropogenic noise research, recognizing the value that both wild and managed-care research plays in illustrating how noise pollution affects welfare including physiology, behavior, and cognition.

## 1. Welfare

Cetacean welfare is a topic of concern for the public, scientists, and policymakers. Welfare is defined as the well-being of an individual, which teeters between two opposing states, positive and negative, as the animal responds to its environment [1]. Each experience is able to tip the scale towards positive or negative outcomes, but welfare status should not be dichotomized as only “good” or “bad” [2,3]. The framework of animal welfare began with the principles of “The Five Freedoms” that include “freedom from” stimuli that initiate negative experiences, such as poor nutrition, poor environmental standards, poor health, negative behaviors, and poor affective states [4]. Welfare science has expanded these freedoms to those of the Five Domains.

The Five Domains approach is composed of a mental state and four interacting physical states, which include nutrition, environment, health, and behavior [5,6,7]. The five domains additionally promote positive experiences through enrichment as well as freedom from negative experiences extending beyond preventing cruelty into positive welfare promotion [5,6,7]. Potential negative experiences, such as pain and suffering, are central concerns to an animal’s quality of life. Negative affective states or experiences can arise from both physical and cognitive stimuli [5]. While pain impacts the physical state of the animal, suffering includes pain plus negative affective states [3]. The impact of negative experiences depends on the intensity and duration of the event, with intensive stimuli possessing the potential to demotivate animals from exploring positive rewarding behaviors, or even attending to basic needs such as food or water [6]. Therefore, it is important to replace negative situational experiences with positive ones to shift the animal’s welfare state into the positive spectrum [6].

Due to their reliance on sound, cetaceans are vulnerable to potential distress in environments where sound exceeds various threshold levels [8], thus exposing them to negative experiences and poor welfare outcomes related to the Five Domains. A negative acoustic environment can affect the other interacting domains. If an environment promoting negative welfare inhibits foraging behaviors by masking echolocation, the animal can suffer poor nutrition, and if the poor nutrition continues, poor health outcomes are experienced. Similarly, a negative acoustic environment can impact mental states due to chronic negative experiences that can impact the four physical domains by causing a chronic stress response.

Both wild animals and those housed in managed-care potentially face acoustic welfare concerns. In managed care, acoustic welfare challenges may be influenced by the habitat itself [2,3], as animals are housed in two main types of environments: pools and natural lagoon enclosures [9]. Reverberations related to the reflective concrete properties in pool environments are suggested by some to be a potential source of noxious acoustic stimuli; however, forethought of pool design may help mitigate noise reflected off smooth concrete surfaces. This could include the use of new multilayer membrane materials that alternate the composition of solid and liquid materials [9,10]. In contrast, while natural features may minimize the reverberations experienced in pool-based aquaria, different acoustic welfare concerns may depend on the geographic location of a lagoon-based facility and the degree of exposure to anthropogenic sound sources, including recreational and commercial vessels [9].

Acoustic welfare in managed care settings remains a poorly studied field. This has prompted regulatory bodies like the USDA/APHIS (Animal and Plant Health Inspection Service) to reference the wild literature to inform welfare standards under managed care, arguing that an understanding of natural behavior and ecology are key to informing appropriate welfare standards for cetaceans under human care. Furthermore, APHIS has, in the past, requested comment from the public on whether they should establish noise thresholds for each species under human care [11], and some groups have been keen to make tenuous claims about captive whale acoustic welfare, based on inappropriate comparisons [12]. While we believe that there are opportunities for both managed-care and wild domains of acoustic welfare science to inform each other, one must accurately assess the conditions of wild animals and compare how they may differ from, and relate to, their facility-based counterparts to provide meaningful recommendations. For example, the noise effects of a distant rollercoaster on a pool of orcas may be fundamentally different than seismic water guns (with amplitudes up to 201 dB re 1 μPa) on animals, with no barrier between them and that extreme sound source [13], as the sound profiles of these stimuli are very different. In other cases, animals in managed-care facilities and wild animals may experience similar acoustic welfare outcomes. This could include cetaceans housed in a sea pen experiencing the same levels of shipping and low frequency noise as other wild cetaceans within the same area. In a welfare context, no one facility type or setting (wild vs. non-wild) should be seen as inherently acoustically superior to another without a comprehensive evaluation of the mitigating factors present related to sound exposure.

Acoustic welfare concerns across both toothed and baleen cetaceans have included extreme physiologically damaging outcomes, including deafness and death [14,15], and have been studied far more extensively than potential effects in managed-care settings. Despite these critical consequences to wild individuals, the discussion of these issues, welfare consequences were more centered around the conservation and preservation of stocks over the experiences of individual animals, with a few exceptions [16,17,18]. Welfare ethicists have expressed concern related to the difficulty of assigning and assessing values of well-being to wild animals outside of human care [2]. However, a welfare framework that centers on the experiences of individual animals may help identify sub-lethal effects on wild cetaceans and allow us to frame the discussion beyond just the ways that noise affects population numbers [18,19].

The next step in managed-care acoustic welfare is to integrate the focus on the individuals under human care with the massive amount of data, resources and advanced techniques garnered for wild studies on anthropogenic noise effects on cetaceans in the wild. Furthermore, we recognize that behavioral data taken from wild studies can inform the welfare of animals under human care who live acoustically connected to the ocean [20]. Conversely, data collected on acoustic welfare in managed-care settings can inform wild studies with respect to acute responses and cognitive effects furthering the opportunity for controlled studies with protocols that are impossible in wild settings [20]. Therefore, we advocate for a synergistic paradigm to evaluate acoustic welfare in cetaceans, both in the wild and under human care such that each can inform the other when considering the acoustic welfare of all cetaceans.

## 2. Cetacean Audition and Auditory Processing

Cetaceans evolved from terrestrial vertebrates that developed specialized ears shaped for sensitivity to their environment. There are three parts to cetacean ears: external ear canals that are fused in multiple species, the middle ear that amplifies sounds, and the inner ear, which is comprised of a cochlea that performs the analysis of spectral characteristics [21]. Historically, two pathways for sound conduction to the ear have been hypothesized: (1) the primary path along the pan bone region of the jaw, which deliver sound to the ossicles of the ear, and (2) an external path through the auditory meatus. Now, toothed whales are believed to conduct sound through the internal mandibular fat body pathways along the jaw [22,23]. These mandibular trumpet-shaped fat bodies have a low structural impedance that might be specialized for capturing different frequency signals, as well as amplifying sound [21]. Once sound has entered the ear, hair cells are stimulated in the cochlear membrane causing receptor potentials, which are communicated to nerve fibers and sent to an expanded auditory cortex for processing [22]. Each part of toothed whales’ auditory systems evolved for sound reception and amplification of sounds traveling in water, making them susceptible to rising levels of anthropogenic noise pollution [24].

## 3. Anthropogenic Noise Pollution

Both wild animals and animals in managed-care living in natural lagoons can face similar anthropogenic noise environments. The Anthropocene ocean is a mixture of biological, geophysical, and anthropogenic sources, and the ratios of each sound type are rapidly changing [24]. Many industries harness sound to map out the ocean to extract valuable resources producing powerful vibrations, while vessels designed for leisure complicate the soundscape. This diversity of sound production generates noise that varies in frequency, amplitude, and potential harm.

### 3.1. Categories of Anthropogenic Noise and Signature Characteristics

Anthropogenic noise is divided into low-frequency (less than 1000 Hz), mid-frequency (1–20 kHz), and high-frequency (>20 kHz) categories [25]. Sources include, but are not limited to, vessel traffic, sonar devices, naval sonar, fish finders, offshore windmills, personal watercrafts, marine animal deterrent devices, dredging, and hydrocarbon drilling [24,25,26]. These sources are produced either continuously, or at intervals, with energy levels varying globally.

Low-frequency sound is the most pervasive anthropogenic sound type, due to the long distances that it travels in uninterrupted deep water [25]. Low-frequency vessel noises are the most abundant sounds contributing to marine noise pollution [25,26,27]. There are numerous low-frequency anthropogenic sound sources, including but not limited to, vessel traffic, seismic exploration for hydrocarbon farming, and dredging [25]. Shipping traffic sound results from numerous sources on the vessel, including the hull of the ship, propulsion machinery, and the cavitation of the propeller. These factors define the individual acoustic signatures that typify each ship, but they are indistinguishable at long distances due to the ability of low-frequency sounds to amalgamate, creating broad spectrum peaks between 5 and 500 Hz [25]. Thus, vessel sounds may create different experiences and affect cetaceans differently, depending on the distance from the vessel, the signature of the ship itself, or the combined effects of the amalgamation of multiple vessel sources. Vessel traffic is a continuous noise source rather than a sharp onset noise. In shallow areas with high presence of vessel activity, low-frequency waves increase. However, shallow-water vessels create more concentrated noise within these coastal regions, ultimately raising ambient noise levels [24]. In areas of high vessel density, some coastal dolphin populations have altered their activity budgets. Indo-Pacific bottlenose dolphins in an urbanized estuary allocated less time for resting and more time for traveling at increased speeds [28]. Consequently, dolphins travelling at higher rates of speed who are allocating less time to rest will need to successfully forage more or suffer an energetic deficit and potentially a lower body condition composition and poorer welfare outcomes [29,30].

Vessels may also come equipped with technologies that produce powerful sounds tied to seismic exploration airgun arrays used in hydrocarbon extraction and sonar arrays used in military and commercial applications. Seismic exploration produces sound from a charged air cannon at high energy levels downwards to probe the seafloor for hydrocarbon extraction. Seismic airgun arrays emit pulses at frequency levels of less than 1000 Hz, with upward frequencies above 15 kHz at sound pressure levels around 240 dB re Pa^2^s [26]. Airgun noises are often loud persistent noises that can penetrate an area for weeks or months [24]. These acoustic tools are a source of concern for many in the marine mammal community, given their power and persistence, and as of 2021, the National Marine Fisheries Service (NMFS) has issued new rules associated with marine mammal take authorizations in the Gulf of Mexico related to oil and gas exploration [31]. These regulations include: standard detection-based mitigation measures, including use of visual and acoustic observation to detect marine mammals and shut down acoustic sources in certain circumstances; a time-area restriction designed to avoid effects to bottlenose dolphins in times and places believed to be of particular importance; vessel strike avoidance measures; and monitoring and reporting requirements. The particular focus on bottlenose dolphins offers an opportunity for captive and managed-care led/funded studies to help inform compliance with this rule, as this species is the most ubiquitous under human care.

Active sonar is produced across multiple sound frequencies and categorized across multiple levels due to their variety of practical applications at different frequency levels. Multi-categorical sonars are used globally at locations that are stationary as well as mobile, as part of ships. Low-frequency active sonars (LFAS) are the most far-reaching sonars that are used for broad surveillance; they emit frequency modulated and continuous wave components at 215 dB per projector, at frequencies ranging between 100 and 500 Hz [25,26]. Consequently, long-ranging low-frequency sounds such as LFAS sonar may impact larger numbers of cetaceans, including geographically isolated stocks, because it travels long distances [32,33]. LFAS may be a fruitful area of research, given its far-reaching nature, ability to affect individual and groups of cetaceans, and the serious repercussions of strong doses [34]. Mid-frequency active sonar (MFAS) used for anti-submarine warfare (ASW) is designed to locate objects at distances of a few hundred meters to a few kilometers. Military sonar exercises linked to multiple mass stranding events in multiple cetacean species and naval operations, including ASW, account for 9% of global beaked whale, *Ziphiidae* sp., stranding events [35,36]. Additionally, ASW exercises can induce temporary hearing loss in toothed whales [37]. It is also important to note that sonar activities are not limited to military applications; commercial sonars operate a narrow downfacing beam or multibeam between 3 and 200 kHz, designed to measure depth and map out profiles of the seafloor [25,38]. Sub-bottom profilers produce sound at source levels as high as 230 dB [38]. Multibeam deep-water mapping systems operate at high sound outputs (245 dB), but are oriented highly directionally [38]. Hydroacoustic sonars, also known as fish finders, primarily operate at 20–1000 kHz range [38]. Much of the focus has been on the effects of military-related sonar applications, while the effects of commercial sonar types are less prevalent in the literature, due to their linear nature and limited operating ranges. However, some commercial active sonar types produce incidental spectral peaks outside of their center frequency that could potentially be detected for hundreds of meters from the source [39]. Future studies could focus on the behavioral effects of commercial sonar, like fish finders and seabed mapping technologies that operate within currently defined safe hearing thresholds.

Unlike sonar and seismic surveys, acoustic deterrent devices (ADDs) and acoustic harassment devices (AHDs) are used to purposefully control the predation behavior of aquatic food stocks. In regions of dense aquaculture, the use of acoustic deterrent devices and acoustic harassment devices is changing the aquatic soundscape and becoming a widespread contributor to anthropogenic noise pollution [40]. ADDs generate omnidirectional pings at lower decibel levels that oscillate between frequencies of 5–160 kHz at 150 dB to avoid habituation [25,38]. AHDs produce higher source level pings and frequency sweeps at 205 dB between 5–160 kHz [38]. Both ADDs and AHDs must avoid habituation responses because they are used to repel marine mammals to reduce loss of stock and by-catch at fisheries, and aboard fishing vessels. Although many of these management devices target pinnipeds, there are ADDs specifically designed to keep cetaceans from becoming by-catch in gill nets [41]. However, exposed cetaceans may suffer from temporary or permanent hearing loss, avoidance of habitat, loss of prey, and masked communication [42].

### 3.2. Factors Influencing Anthropogenic Noise

Locations around the globe have varying compositions and rates of anthropogenic noise production. Within the last 50 years, ambient low-frequency sound increased as much as 32-fold along shipping routes, but low-frequency noise has not increased at the same rate globally [24]. In the Northwest Pacific, low-frequency noise has increased at a rate of 3 dB/decade over 60 years with the Indian Ocean not far behind varying at a growth rate of 2–3 dB per decade. In comparison, the Southeast Atlantic, Northeast Pacific, and Equatorial Pacific have shown slight decreases in low-frequency sound at the seafloor [43]. Additionally, areas of dense population and traffic close to the coast have higher ambient noise levels of low-frequency sounds [24]. While quieter shipping technologies have led to a decrease in areas (like the South Atlantic), where shipping rates remained constant, the dominant sound sources are now mid-frequency seismic air guns [43]. Finally, in high tourism regions, scenic vessels may pass dolphin populations as much as every 6 min during daylight hours [44].

The propagation of anthropogenic noise varies by the depth of water, and the intensity of sound depends on the geophysical constitution of the location that it is produced in, as well as its source. In shallow water, there is greater noise pollution due to greater reflection off substrate and the shallow longitudinal wavelengths of low-frequency sounds. In noisy areas of the Ganges River, vessel sound pollution increased the ambient noise levels by 14 dB, with an average water column height decrease of 1.5 m during the dry season [45]. Although deeper natural river channels may act as a buffer to some of the increased vessel noise during times with low water levels, river dolphins require full use of shallow and deep areas for resting and foraging [45]. During louder times, marine cetaceans may retreat into deeper waters, but river dolphins and cetaceans restricted to shallow waters in sea-side lagoons or sea pens may not have such opportunities.

In human care facilities, anthropogenic noise composition depends on multiple factors including, but not limited to, facility type, amount of exposure to the ocean, life support machinery, presence of shows, and location. Facilities that house marine mammals in pools do not have additional oceanic anthropogenic noise, but their enrichment devices and life support systems impact ambient noise levels [9]. Enrichment components such as wave machines, sprinklers, and bubble machines produce extra environmental noise from machinery and action. The life support systems contribute the most to structure-borne ambient sound with continuous noise produced from pump machinery [46,47]. Results on the decibel and frequency of life support systems have been mixed. One study found that ambient noise levels in the largest pool of the Monterey Bay Aquarium were 15–25 dB higher than the bay environment that it was simulating, increasing with proximity to the pump room [46]. More recently, research at the Georgia Aquarium dolphin exhibit found that overall noise levels in-air and underwater were minimally above lower hearing thresholds of bottlenose dolphins, *Tursiops truncatus*, and the fully operational (all pumps running) life support systems raised ambient noise levels by approximately 10 dB primarily in frequencies under 1000 Hz [47]. Ambient noise levels above 1000 Hz were not significantly different, with the life support machinery completely on or off, and ambient noise levels of 15,000 kHz near the bottlenose dolphins’ most sensitive hearing range exhibited no marked impact due to life support systems [47]. This indicates that life support systems may not be as much as a noxious sound source or initiator of negative affective states that lead to poor welfare, however, the sampling of soundscapes across facilities to ascertain noise levels is suggested to monitor acoustic welfare.

Additionally, sound can be introduced by external speakers outside of the pool, as well as through underwater speakers. Some pool facilities use sound and music in public demonstrations, but those auditory soundtracks and special effects of shows did not contribute sound above life support system levels when speakers were not in the vertical plane of the water [47]. Given that dolphins participating in demonstrations spend a large portion of the show with their ears above water, this potential area of impacted welfare should be studied further. Each show has a different sound level, acoustic environment, and soundtrack selection that impact the composition of ambient levels. Managed-care facilities should also measure the sound levels at the locations where the dolphins are stationed to evaluate sound levels for the purpose of avoiding noise levels near temporary hearing shift-inducing zones (see Section 3.3 below). While anthropogenic sounds can certainly be damaging to marine mammals, introduced sound does not necessarily need to be thought of only as aversive. Through partnerships with scientists familiar with cetacean communication, acoustic enrichment opportunities exist (see Signature Whistle Playbacks).

Facilities that are exposed to oceanic anthropogenic noise vary in composition, as some are pool facilities close to noise sources and others are lagoon facilities, with varying degrees of sonic exposure to the ocean. Some protected lagoons may experience less ambient oceanic noise than netted seaside sea pens that are not tucked behind natural or artificial barriers, as netting cannot buffer environmental acoustic noise. The ultimate determinant of acoustic welfare must consider many factors, including the ambient noise, as well as the habituation profiles that the animals display toward it. Hypothetically, a coastal lagoon-type facility near an industrial shipping channel with random exposures to low-frequency sonar pings may have greater welfare issues than an open sea pen with little surrounding vessel traffic. It all depends on the relatively unpredictable nuances of the environment. One cannot broadly say that one habitat is more ideal than another, without testing this from an acoustic and welfare perspective. Furthermore, constant predictable exposures outside of hearing thresholds may be habituated readily and may have minimal welfare consequences. Sound exposures will vary by time of year, predictability of vessel traffic, depth of waters, and location; therefore, each facility will experience unique ambient acoustics. Intensity of tourism and the predominant types of vessels around the facilities will also impact ambient noise in coastal facilities.

### 3.3. Current Regulations

Current anthropogenic noise policy development uses many of the same sets of data and sources. For example, the Danish Center for Environment and Energy uses criteria established by Southall et al. [48] and Southall et al. [49] to define detrimental Type-I sounds as very fast onset with a short duration and large bandwidth, and less damaging P-Type sounds as typically narrow-bandwidth signals and species are assessed based on sound type [50]. Although, more recently, there have been calls to abandon the categorization of “pulse” and “non-pulse” stimuli, as sounds can have different characteristics at source and at distance, and replace this with sound classifications organized by industry type [20]. In the United States, the Marine Mammal Protection Act (1972) prohibits the purposeful and accidental harm or killing of marine mammal species [51]. The National Oceanic and Atmospheric Administration produces guidance on mitigating the effects of anthropogenic noise activities [52]. Their efforts are designed to prevent aquatic species are exposed to certain sounds that might harm monitored stocks [48]. Currently, there are three proposed cetacean hearing sensitivities: low-frequency, high-frequency (which is the most abundant group including bottlenose dolphins and killer whales), and very-high-frequency [48]. Noise exposure criteria are based on potential to induce temporary hearing loss or temporary threshold shifts (TTS) in behavioral trials. Groups are weighted with auditory functions and generic band-pass filter equations, then TTS onset is extrapolated via exposure functions. Permanent threshold shifts (PTS), or permanent hearing loss, are estimated from TTS growth rates [48]. The low-frequency and high-frequency groups’ initial TTS onsets are nearly identical at 178 and 179 dB re 1 μPa^2^s under water, while the temporary hearing loss threshold for the very-high-frequency group is lower at 153 dB re 11 μPa^2^s under water. Onset of PTS is 20 kHz above the initial start of TTS. High-frequency and very-high-frequency groups experience PTS onset at 198 and 199 dB re 1 μPa^2^s, while the very-high-frequency group’s threshold is lower at 173 dB re 1 μPa^2^s (see Table 1 for noise thresholds for common species in managed-care).

From the TTS and PTS onset levels for each hearing group, potential welfare impact by hearing damage can be measured. However, group criteria are not based on cognitive impact, and there are species within the groups that have not been studied individually. In beluga whales, bottlenose dolphins, harbor porpoises, and finless porpoises, hearing sensitivities have been studied extensively. Approximately one third of the high-frequency hearing group have been measured for hearing sensitivities and, in contrast, only three species of very-high-frequency cetaceans’ (*Phocoena phocoena, Neophocaena phocaenoides,* and *Kogia breviceps*) hearing sensitivities have been measured, with each species indicating large differences in their upper frequency hearing limits [48]. Additional research on the very-high-frequency group’s individual species’ upper threshold limits and peak hearing sensitivity would greatly aid the study of acoustic welfare and anthropogenic noise impact.

Recently, there has been a reevaluation of criteria used for assessing the severity of behavioral responses and predicting effects of anthropogenic sounds in animals exposed to anthropogenic noise, especially in the experimental domain. Originally not considered separately, response scores of cetaceans in managed-care and wild settings have been decoupled into multiple severity scales [20]. Under this paradigm, scores for managed-care subjects would be divided into tracks corresponding to responses that include trained behaviors, such as stationing, and untrained behaviors. Focusing on the effects of anthropogenic noise during trained behaviors provides a method to measure performance and the level that a noxious stimulus will supersede positive reinforcement [20]. All managed-care behavioral responses range from 0—no response, 1—just detectable response, 2—aversive or negative response, 3—aversion, to 4—sensitization, accounting for habituation (See Cognitive Issues section for more on habituation and sensitization effects) [20]. Wild animal disturbance, in contrast, would be scored on severity across three domains: survival, reproduction, and foraging. These disturbance effects range as scores from 0 (no response) to 9 (serious injury or mortality, exhaustion of energy sources, or failure to successfully reproduce during breeding season), not assuming habituation effects [20]. These response scales are intended to assess discrete exposure events, and therefore are NOT valid for evaluating chronic noise exposure outcomes. Coupled with threshold shifts, earlier versions of these severity assessment scores have been historically used to create step-wise threshold levels and broad taxa-based regulatory approaches [48,49], however there is movement away from broad all-or-nothing threshold categories and broad taxa based approaches, due to the difficulty of obtaining a sense of the welfare consequences of noise at the individual and species level, as well as the difficulty of assessing the role context plays when predicting consequences of discrete noise exposures [20]. Southall et al. (2021) consideration of multiple species in wild settings also applies to animals in managed-care in that facilities with mixed species groups can mitigate noise by adjusting acoustics stimulation to the thresholds of the most sensitive species.

## 4. Consequences of Anthropogenic Noise

### 4.1. Sub-Lethal Physiological Changes

Although temporary hearing shifts are limited in TTS zones, frequent sound levels from 17 dB to 50 dB above TTS hearing thresholds induce PTS for cetaceans in all hearing groups [48,50]. Twenty to thirty minutes of pulsating sounds at 173 dB were strong enough to induce TTS in wild bottlenose dolphins [53], which is lower than the high-frequency group’s TTS onset indicated above in the most recent extrapolated TTS. Mid-frequency active sonar produced at 203 dB induced TTS in bottlenose dolphins in as soon as 5 min of exposure [37], suggesting that the level of intensity above the safe hearing threshold impacts how quickly threshold shifts occur, and the duration of time may change the upper limits of safe hearing thresholds. Studies have also found that TTS can be induced at lower levels in beaked whales than previously reported for bottlenose dolphins [54]. Additionally, potential hearing shifts (i.e., TTS or PTS) depend on the positionality of the animal in respect to the sound source. Sounds between 2–30 kHz in bottlenose dolphins affected hearing thresholds differently depending on the location sound source, indicating directional sensitivities to sound sources, and the position of the cetacean’s head directed 180° away with their tail facing the sound source may provide some respite from aversive sounds [55]. This angular sensitivity to sound indicates that potential harm is affected by the incidental position of animals, and may be further complicated by sound that is stemming from multiple sources around whales or dolphins. This complexity presents an additional challenge to assessing anthropogenic noise induced harm among wild individuals, whereas their maintained counterparts in facilities can provide additional insight into this phenomenon through behavioral studies focusing on the way in which cetaceans orient their heads in respect to certain anthropogenic sounds.

Hearing loss potentially causes changes in echolocation rates and click frequencies, depending on which frequencies are lost to the cetacean’s perception of sound. For echolocation to be successful, dolphins must be able to produce click trains and be able to receive those clicks back to process. Bottlenose dolphins with high-frequency hearing loss lower their click emission energy into frequency ranges that they can hear, and they increase how loud their clicks are [56]. Upper frequency loss in individuals was correlated with an approximate 0.2 kHz decrease in click center frequency for every 1 kHz of upper frequency lost. Each dolphin altered their individual click parameters and temporal click emissions in a different unpredictable pattern from other individuals with upper frequency hearing loss; the extent of hearing loss was not a predictable indicator of how much the dolphins reduced their click frequency [56]. No longitudinal studies have been performed focusing on adaptation of echolocation parameters on members of the high-frequency group with reduced upper hearing thresholds. Similarly, echolocation changes among individuals with upper frequency loss in the very-high-frequency group have yet to be explored. The effect of altered echolocation parameters on foraging success is unknown. Impaired hearing and reduced echolocation processing capabilities could dramatically affect the fitness of an impaired wild cetacean if those constraints impact one or more of the five domains of welfare. For example, altered acoustic parameters of echolocation and reduced hearing may limit foraging success and navigation, leading to poor affective states relating to nutrition, environment, and health domains.

Additionally, stranding events are potential repercussions of hearing loss. Ketten (1995) analyzed potential repercussions of blast charges to cetacean ears, and predicted the repercussions of multiple levels of acoustic trauma, including hearing loss, mixed lethality zones, and death [33]. Mann et al. (2010) found that in three species of cetaceans (bottlenose dolphins, rough-toothed dolphins, short-finned pilot whales) some individuals showed significant hearing threshold shifts equivalent to severe (70–90 dB) or profound hearing loss (>90 dB) in humans [57]. However, Mann et al. (2010) also recorded multiple stranded cetaceans without hearing loss. Consequently, more research is needed on stranding events investigating hearing threshold shifts and tympanic tissues in stranded toothed whales. Stranding events lead to serious physiological repercussions that often lead to death without intervention.

### 4.2. Stress Effects

In the wild, and in certain experimental conditions in managed-care facilities [13], anthropogenic noise increases stress markers and stress effects. In the Ganges River, the cost of chronic anthropogenic activity decreased caloric intake by as much as 40% in noisy conditions, increasing metabolic stress on the endangered South Asian river dolphin populations, *Platanista* sp. [45]. Amplified anthropogenic sound intensified the metabolic deficit, doubling metabolic cost during times of quadrupled ambient noise levels [45]. Tagged whales exposed to controlled levels of low-frequency active sonar displayed reduced rates of deep diving associated with foraging, and exhibited potential extra metabolic stress [58]. When exposed to intense levels of seismic gun sources (>100 kPa), captive belugas exhibited increased epinephrine and norepinephrine stress hormones [13].

Of course, acoustic welfare is not limited to the application of sudden or startling noises. Sometimes, we see decreased stress responses through the removal of a typically perpetual noise in what is a sonically intense environment. After the events of 11 September 2001, the Bay of Fundy experienced a 6 dB drop in low-frequency noise levels. North Atlantic right whales, *Eubalaena glacialis*, responded to the decrease in ambient sound levels with a significantly decreased level of expressed stress hormones in their fecal matter [59]. The Anthropause associated with the 2020 COVID-19 pandemic is another rare occasion of decreased human activity across the globe that has led to altered oceanic soundscapes and the opportunity to further study how chronic noise is affecting wild populations, including geographically isolated stocks, as well as the potential benefits of a repose from certain perpetual noises, such as cruise ships and other low-frequency vessel noises [60,61].

In the managed-care welfare landscape, acoustic stress effects are probably one of the issues most conjectured about by opponents of the presence of animals under human care [12,62]. Papers from authors that discuss noise and stress struggle to separate acute versus chronic sources in captive settings, and often make broad comparisons to distantly related species [63]. Given the unique nature of cetacean hearing physiology and anatomy [64], and the limited amount of data that we have on what sounds that these animals are exposed to in a broad sense, this remains an open area of welfare research. This is especially true given that multiple studies have shown that baseline cortisol levels in captive bottlenose dolphins are no higher than in their wild counterparts, which could have implications for hypotheses related to chronic exposure and potential habituation processes [65,66,67,68].

### 4.3. Acoustic Behavior and Masking Effects

Many anthropogenic noises fall within the range of cetaceans’ communication, causing masking effects that could indirectly reduce individual fitness. Baleen whales are especially vulnerable to this acoustic masking by low-frequency sound sources, due to their reliance on long distance communication to attract mates and repel competing males. Humpback whale, *Megaptera novaeangilae*, song primarily consists of frequencies below 1000 Hz, but they can reach upper harmonics in the range of 24 kHz [69]. The lower frequency notes of humpback song that are used to increase that animal’s fitness propagate across long distances, and are attenuated or outright canceled by overlapping noise sources, such as vessel traffic, LFAS, and hydrocarbon exploration [69,70,71]. High-frequency hearing groups experience acoustic masking and echolocation shadowing (i.e., areas of signal masking due to acoustic interference) of lower frequency and mid-frequency sounds too. For very-high-frequency species living in the Ganges River, cavitation noises produced by vessel traffic completely shadowed the broadband clicks of foraging dolphins [45].

Cetaceans primarily use two strategies to counter acoustic masking: (1) they increase the amplitude of their vocalizations (Lombard effect) and (2) they shift signal characteristics of their vocalizations. These responses increase the likelihood that the callers are heard through increased volume (1) or occupying a frequency with less spectral clutter (2). In areas saturated with noise pollution, many species of cetaceans exhibit the Lombard effect, which is an increase in the volume of vocalizations in the presence of excessive noise [72,73]. Bottlenose dolphins in Sarasota Bay, Florida responded to temporary increased sound bouts by increasing the intensity of their overall vocalizations, but the shift was not congruent among signature and non-signature whistles [73]. The dolphins shifted the intensity of their signature whistles less than their non-signature whistles [73]. Bottlenose dolphins respond to whale watching vessels by increasing the frequency of calls with an upward shift by up to 1.99 kHz [72]. Separate populations of killer whales of the Puget Sound and off the coast of Iceland responded to potential masking risks by calling louder [72,74]. Some species battle acoustic masking by fundamentally altering the frequency and duration of their calls. In addition to the Lombard effect, killer whales also responded to vessel noise by modulating their vocalizations, by increasing the durations of their calls [75]. Over just a few decades, right whales have shifted their calls to higher frequencies and decreased calling during peak background activity [76]. When exposed to vessel, naval, and air gun noise pollution of right whales, fin whales, *Balaenoptera physalus*, and grey whales, *Eschrichtius robustus*, change fundamental spectral characteristics, including the frequency components of their calls [76,77,78]. These responses to acoustic masking may have an indirect fitness reduction on species, and not every species responds the same. Although these changes may not be large percentages in the total hearing and vocal production range of cetaceans, shifts in the frequency of whistle production decrease the propagation of calls, and may decrease the efficacy of communication increasing energetic efforts, impacting the nutrition and health domain.

Some populations of cetaceans respond to the increased presence of noise pollution by changing their vocal behavior, including the modification of vocal production rates. Moreover, they display avoidance tactics. Changes in vocal production rates have been marked across many species. Beaked whales and right whales reduce call rates in high noise conditions. When harassed by operating vessels near Sado Estuary, Portugal, bottlenose dolphins mean overall call rates decreased, and the dolphins displayed significantly reduced click rates [79]. Bottlenose dolphins off Sarasota Bay, Florida, are exposed to vessels passing as close as 100 m every six minutes. When approached by vessels, these dolphins temporarily increased whistle production, then call rates decreased after the vessels left [44]. Disruptions in communication may possibly affect bonding, social structures, learning opportunities provided by mother to calves, and successful hunting, all of which in turn jeopardize the health of populations. Smaller cetaceans that are within the very-high-frequency hearing group have a shorter threshold for noise exposure, and it is not unreasonable to surmise that they experience increased negative welfare outcomes, due to increased levels of anthropogenic noise. Heaviside’s dolphins, *Cephalorhynchus heavisidii*, relax acoustic crypsis (an anti-predation acoustic modification where the sound producer operates at a higher or lower frequency than is detectable by predators) to increase echolocation range which may expose them to higher risks of predation by eavesdropping predators [80]. This reduction in acoustic crypsis may be further impacted by increased levels of anthropogenic noise. Studies on the impact of anthropogenic noise on very-high-frequency species kept in captivity, such as Commerson’s dolphins, *Cephalorhynchus commersonii*, would provide instrumental information for shy wild species that are difficult for researchers to study. For example, acoustic crypsis in the presence of anthropogenic noise may be studied in Commerson’s dolphins by setting up a simple playback experiment. Potential experiments focusing on acoustic welfare in very-high-frequency species using a playback methodology could focus on respiration rates (a common dependent measurement used in facilities to assess increased metabolic function due to potential stress), displacement from the sound source, or other welfare metrics.

Rose and Parsons (2019) speculated that the influence of smooth walls and increased reverberations may cause dolphins to reduce rates of echolocation [81]. In this case, the argument is that the reverberations will cause dolphins to reduce their use or change the parameters of their echolocation. To date, this concern remains conjecture, as it has not been systematically assessed. To address this issue, a fruitful area of research might involve the question of masking. Investigations on any potential decrease in echo-rates under managed-care could compare the levels of echolocation between pool facilities and lagoon or sea pen facilities, where environments are less likely to cause possible masking reverberations. Research on echo-rates could be done under experimental conditions if one was concerned about habitat familiarity inhibiting echolocation in managed care pool settings. A match-to-sample design could be used to determine echolocation rates, where the subjects would be incentivized to use echolocation to solve the task, and variations on the willingness to use the sensory system could be interpreted as a potential acoustic concern in that habitat. It would be difficult to isolate whether reverberations or masking explain any decrease in echo production, but the research on production levels should be conducted first before assuming negative acoustic welfare in pool facilities based on currently available data, or the lack thereof. Furthermore, reduced echolocation rates are not necessarily a negative welfare outcome. Toothed whales have some control over self-sound exposure beyond just ‘echolocate’ and ‘no-echolocate’. Just as children are taught not to yell inside, dolphins too likely possess the ability to learn to modulate their signals to prevent their own negative welfare consequences. This ability should be considered when evaluating acoustic welfare as a function of echolocation production.

### 4.4. Alterations in Behavior

One of the most common behavioral responses to anthropogenic noise includes avoidance behaviors and altered distribution at times of increased anthropogenic noise presence. Bottlenose dolphins in the Dolphin Bay of Bocas del Toro archipelago are subjects of the largest whale watching operation in Panama. When exposed to vessel noise, the dolphins in Dolphin Bay abruptly changed activity states, which led to decreasing amounts of foraging and increased energy expenditures from avoiding the stimuli [82]. Bottlenose dolphins in the Spanish Mediterranean Sea preferred to inhabit areas of low recreational activities, however, the dolphins remained a large presence in areas where fishing-related activities persisted [83]. These patterns suggest that the dolphins prefer to be away from increased anthropogenic activity, but they are willing to experience human-made sound stresses when foraging is necessary. Dolphins in the Sicily Strait also follow fishing trawlers in this specialized foraging strategy [84]. Distribution changes in the water column, known as dive shifts, have been explored in multiple cetacean species, with each species responding using distinct strategies for each anthropogenic sound source. When exposed to LFA sonar, cetaceans that spend much of their time at the surface, such as killer whales and pilot whales, *Globicephala* sp., continue their shallow diving behavior and cease any deep diving foraging they would normally exhibit [58]. Sperm whales, *Physeter macrocephalus*, normally spend much of their time in dives foraging for large squids that live at deep depths. When exposed to LFA sonar, sperm whales’ dives also become abnormally shallow, which reduced time spent foraging, and long periods of exposure could lead to increased metabolic stress resulting in reduced body condition and increased stress to populations [58].

Unfortunately, right now, we simply lack enough data to support many conclusions about the behavioral effects of external sounds in a managed care environment. However, one study on bottlenose dolphin social play found a reduction in play frequency during bouts of pool-adjacent construction work but not frequency of agnostic or sexual interactions [85]. Expanded studies on dolphin social play could be used to measure the degree of impact on behavioral responses to external anthropogenic sounds. Additionally, we can use surfacing behaviors or respiration rates described in the wild literature as metrics for welfare in managed-care settings. We have used surfacing inhibition behaviors in managed care settings to determine aversion to specific drone frequency playbacks in air (Bruck et al. unpublished). If the concern is that noise from music or fireworks during a public exhibition of the animals is aversive [12], then animal respiration rates should change. Respiration rates were used as a welfare measurement during some of the first captive playback sessions with signature whistles in the late 2000s [86]. If sound sources are localized, then one could evaluate habitat utilization as means to determine if sound stimuli are noxious. Since most facilities do not, as a rule, have an ‘ear’ on their animals, localized noxious noise stimuli may go undetected. Facilities could invest in hydrophones or use tracking tools to monitor their animal’s habitat use, to monitor these impacts, or partner with researchers to help in this assessment. This could be especially true if the stimuli are inconsistent, which makes these types of noises hard to habituate to from the animal’s perspective, and less likely to be noticed by marine mammal specialists and divers who may be on fixed schedules.

### 4.5. Cognitive Issues

Multiple noise exposure events may have a non-linear effect on cognition and behavior due to habituation and sensitization effects. Sensitization manifests as a heightened response that increases each time the individual is exposed to the provoking stimulus [87]. Both the processes of habituation and sensitization interact to produce behavioral plasticity, with arousal invoking stimuli more likely to lead to sensitization [87,88]. While sensitization is a marked increase in response, habituation is characterized by a decrease in response to repeated stimuli presentations. Both habituation and sensitization to anthropogenic sound could be either beneficial or detrimental depending on the sound source because of each type of sound’s varying characteristics including frequency, duration, and intensity. Habituation response to sound might aid the individual by reducing distractions from survival critical behaviors and positive experiences, but it could also reduce survival responses to potential dangers. While a certain degree of sensitivity may aid a whale or dolphin in the avoidance of potential dangers, the added energetic expenditure and distraction from positive and rewarding experiences is a maladaptation. The degree to which these effects develop differ based on an individual cetacean’s experience with anthropogenic noise pollution sources. In the wild and in certain natural lagoon managed-care facilities, it is possible that cetaceans experience potentially startling external sounds. These potentially repeated, startle provoking stimuli could lead to habituation or sensitization [88].

Recently, there has been some attention from researchers working primarily in the wild domain on the cognitive effects of anthropogenic noise. Southall et al. (2021) placed sensitization responses into the most severe spectra of behavioral response severity scores. Sensitization responses in managed-care settings may manifest in different ways depending on whether the dolphin is exposed to discrete anthropogenic noise in a training paradigm or not [20]. Sensitization responses during trained behaviors include: (1) breaks in stationing or avoidance of stations, (2) cessation of current activity to attack or charge sound source, (3) refusal to perform tasks over time even when offered primary reinforcement, (4) repeated aggressive episodes targeting trainers, conspecifics, or objects, (5) failure to recall when logging or in placement of bottom of the pool, and (6) retreating into a refuge space when available [20]. In untrained paradigms, sensitization behaviors include: (1) repeated displacement events towards subordinate conspecifics, (2) acts of aggression toward the sound sources and displacement of objects in the way, (3) retreating into a refuge for more time than the exposure of the sound, (4) negative anticipatory behavior, and (5) logging at the surface or bottom of their enclosure [20]. Southall et al. (2021) represent a framework for evaluating the problem but did not provide information on the pervasiveness of these types of responses. This framework can be expanded by focusing on sensitization/habituation responses in managed-care settings, where caregivers know the animals and the subjects can be revisited. Furthermore, while Southall et al. (2021) address a basic type of learning, researchers can further investigate cognitive effects by focusing on attention, complex learning, and memory with this paradigm. Preliminary data from an acoustic playback response experiment in bottlenose dolphins housed in inland facilities and coastal facilities indicated habituation and sensitization profiles that were unique for each anthropogenic noise [89]. When exposed to LFAS, dolphins housed in coastal facilities responded with increased look duration to the sound source, demonstrating a possible sensitization effect of LFAS [89]. Each anthropogenic sound has the possibility of affecting attention in a unique way because of inherent distinct characteristics of the sound itself (e.g., frequency, amplitude, modulation patterns, and duration). We advocate that future research utilize animals under managed-care to address the question of noise and cognition especially as it potentially relates to survival and reproduction, as well as sociality and cooperation in wild animals [86,90,91].

Cetaceans may respond in a different manner over time due to the plasticity of the habituation and sensitization pathway. In Sarasota Bay, Florida, dolphins exposed to vessel noise waited for vessels to pass before resuming whistle production [44]. Vibratory pile driver noise produced at 140 dB was sufficient to cause cognitive distractions in Naval dolphins asked to perform an echolocation task, resulting in significant decreased target detection [92]. Additional cognitive impacts on learning and memory should be explored, including long-term studies of chronic noise exposure, as it affects the survivability and reproduction of these animals. For example, bottlenose dolphins learn hunting techniques as well as signature whistles of conspecifics, and remember the signals of social partners for decades [86]. Loss of attention can impact more than just foraging success or energy expenditures; dolphins rely on learning and memory to pass on survival critical behaviors. Fear generated from the same startle-invoking stimuli that lead to habituation/sensitization response may also cause pessimistic cognitive biases in non-human animals [93]. These ‘pessimistic biases’ result in greater aversion to ambiguous information and greater amounts of avoidance behavior similar to anxiety behaviors in humans [93]. They may also impact decision making pathways, creating physiological markers of stress due to stress’ role of preparing the individual to respond to potential threats [93]. Although this is an adaptive response in normal states, chronic risk aversion and sensitization to anthropogenic noise can impact fitness significantly, leading to even more stress and negative welfare. The impact of anthropogenic noise on cognition in marine mammals lacks in-depth and controlled studies and management of cetaceans would benefit from increased noise impact and vigilance studies.

## 5. Monitoring Soundscapes in Managed Care 

Passive acoustic monitoring systems, or PAMS, are a vital tool in the monitoring of ambient noise levels (SPLs), acoustic response, and distribution changes of wild stock. PAMS have been used in conjunction with open-source programs such as PAMguard for over a decade [94]. PAMS are a powerful tool for researchers monitoring anthropogenic noise levels and acoustic responses in wild populations [95]. While recording and documenting soundscapes in both facilities and nature are invaluable, PAMS record without sending warning of any changes in the communication characteristics or anthropogenic noise levels of monitored facility’s population. Recently, Jones et al. [96] introduced an open-source Welfare Acoustic Monitoring System, WAMS, that monitors and alerts husbandry staff to sudden onsets of large quantities of signature whistles, which may indicate instances of negative experiences. WAMS provides a flexible interface for acoustic monitoring and is customizable for each distinct facility being studied [96]. An automated system of acoustic monitoring is the next step, but the system needs to be able to recognize types of vocalizations and baseline vocalizations for each facility. Each facility has different soundscapes; some have high levels of signature whistle production, while others are quieter with few or almost no signature whistles [97]. For WAMS to be the most beneficial to researchers and husbandry staff, it is necessary to understand the unique vocal characteristics of each facility in which it will be deployed. Calibration to each facility’s desired vocalization level, acoustic environment, species, and vocalization types of interest are important for the proper functioning of WAMS [96]. If WAMS could be programmed to alert facilities to instances where external sounds are above safe hearing thresholds before noise is capable of inducing TTS and combined with WAMs application to monitor for sudden onsets of signature whistles, WAMS could alert facilities to potentially negative welfare outcomes and be a powerful tool in the welfare management of cetaceans or other species sensitive to anthropogenic noise.

## 6. Conclusions

Anthropogenic noise pollution is an intense and complex challenge that wild and possibly captive cetaceans face. The frequency of anthropogenic noise is increasing with the expansion of human activities causing wakes of effects on cetaceans. Because cetacean ears were adapted to conditions that were presumably quieter than current conditions and are quite sensitive, excessive levels of anthropogenic noise can have deleterious consequences, including death, hearing loss, acoustic masking, behavioral changes including acoustic behavior, and cognitive effects. Cetaceans rely on proper acoustic functioning to maintain social bonds and good body condition; thus, they are at an increased risk of poor welfare outcomes, due to the pervasive noise around them. Whales and dolphins display resilience and behavioral plasticity in response to many aspects of human sound production, yet many populations are still in decline [98,99,100,101]. Some areas of anthropogenic sound effects are well studied, such as hearing threshold shifts, the Lombard effect and acoustic masking, while little is known about the effects of anthropogenic noise on attention, learning, and memory for either captive or wild groups. Habituation profiles from preliminary data suggests that anthropogenic noise exposure does not uniformly affect attention, and possibly other aspects of cognition including learning and memory. Habituation and sensitization responses to external sounds in managed-care and wild settings should be considered when managing acoustic welfare. Future studies involving anthropogenic noise should focus on managing sources of poor acoustic welfare, stress effects based on heart rate or hormone levels, behavioral plasticity, cognitive effects of each anthropogenic noise type, and the behavioral biases such as avoidance of areas with high levels of anthropogenic noise and changes in swim patterns an individual cetacean has developed from exposure to noise related to human activity. Only when we have enough information on each sound type’s harmful effects on cognition and behavior will we be able to create robust responses to mitigate anthropogenic sound, reduce negative welfare outcomes relating to the Five Domains, and promote a positive acoustic welfare environment.

## Figures and Tables

**Table 1:** Exposure limits of cetacean species commonly exhibited under managed-care, including high-frequency (HF) and very-high-frequency (VHF) groups. Proposed sound exposure limits are weighted auditory frequency, expressed as dB re 1 μPa^2^s, and adapted from Southall et al. (2019). Additional species thresholds and auditory weighting information is available in main text.

Species	Frequency Group	Type I Sounds (dB re 1 μPa^2^s)		Other Sounds (dB re 1 μPa^2^s)	
		TTS	PTS	TTS	PTS
*Cephalorhynchus commersonii*	VHF	140	155	153	173
*Delphinapterus leucas*	HF	170	185	178	198
*Globicephala macrorhynchus*	HF	170	185	178	198
*Lagenorhynchus obliquidens*	HF	170	185	178	198
*Orcinus orca*	HF	170	185	178	198
*Phocoena phocoena*	VHF	140	155	153	173
*Steno bredanensis*	HF	170	185	178	198
*Tursiops truncatus*	HF	170	185	178	198
